# Neuroprotective potential of avicularin in mitigating chronic unpredictable Stress‐Induced Cognitive Deficits

**DOI:** 10.1002/alz70859_096424

**Published:** 2025-12-25

**Authors:** Girdhari Lal Gupta, Nikita Patil

**Affiliations:** ^1^ SVKM'S NMIMS University, Shirpur, Dhule, Maharashtra India; ^2^ Department of Pharmacology, Shobhaben Pratapbhai Patel School of Pharmacy & Technology Management, SVKM’S NMIMS Deemed‐to‐be University, Mumbai, Maharashtra India

## Abstract

**Background:**

Chronic Unpredictable Stress (CUS) is a widely used model to induce cognitive impairments and neurodegeneration through the activation of the kynurenine (KYN) pathway. Long‐term stress causes the production of more cortisol, which activates microglia and raises quinolinic acid (QA). This lowers levels of brain‐derived neurotrophic factor (BDNF), which causes neurodegeneration. Avicularin, recognized for its antioxidant and antidepressant properties, was evaluated for its neuroprotective effects against CUS‐induced cognitive impairments.

**Method:**

Mice (Swiss albino) exposed to CUS for five weeks were administered avicularin at doses of 50, 100, and 200 mg/kg from 4^th^ week four to 7^th^ week. Behavioral assessments, including the forced swim test, sucrose preference test, Morris Water Maze, and Passive Avoidance Test, were conducted in the 6^th^ th and 7^th^ weeks. Biochemical analyses evaluated corticosterone, oxidative stress markers, and neurotransmitter levels (serotonin and norepinephrine). The proinflammatory indicators TNF‐α, IL‐6, IL‐1β, and acetylcholinesterase (AChE) activity were measured, and immunohistochemistry evaluated BDNF and amyloid‐beta levels.

**Result:**

Avicularin treatment significantly improved cognitive performance, reduced corticosterone levels, and mitigated oxidative stress. Mice treated with 100 and 200 mg/kg doses showed elevated serotonin, norepinephrine, and BDNF levels, with concomitant reductions in amyloid‐beta levels, inflammatory cytokines, and AChE activity.

**Conclusion:**

Avicularin demonstrates significant neuroprotective effects against CUS‐induced cognitive impairments, reducing neuroinflammation, and enhancing neurotrophic support. These findings underscore its potential as a therapeutic agent for stress‐related cognitive dysfunctions.

